# AtRTD – a comprehensive reference transcript dataset resource for accurate quantification of transcript‐specific expression in *Arabidopsis thaliana*

**DOI:** 10.1111/nph.13545

**Published:** 2015-06-25

**Authors:** Runxuan Zhang, Cristiane P. G. Calixto, Nikoleta A. Tzioutziou, Allan B. James, Craig G. Simpson, Wenbin Guo, Yamile Marquez, Maria Kalyna, Rob Patro, Eduardo Eyras, Andrea Barta, Hugh G. Nimmo, John W. S. Brown

**Affiliations:** ^1^ Informatics and Computational SciencesThe James Hutton InstituteInvergowrieDundeeDD2 5DAUK; ^2^ Plant Sciences DivisionCollege of Life SciencesUniversity of DundeeInvergowrieDundeeDD2 5DAUK; ^3^ Institute of Molecular, Cell and Systems BiologyCollege of Medical, Veterinary and Life SciencesUniversity of GlasgowGlasgowG12 8QQUK; ^4^ Cell and Molecular SciencesThe James Hutton InstituteInvergowrieDundeeDD2 5DAUK; ^5^ Max F. Perutz LaboratoriesMedical University of ViennaDr Bohrgasse 9/31030ViennaAustria; ^6^ Department of Applied Genetics and Cell BiologyUniversity of Natural Resources and Life SciencesMuthgasse 181190ViennaAustria; ^7^ Computer Science Department1422 Computer ScienceStony Brook UniversityStony BrookNY11794‐4400USA; ^8^ Computational GenomicsUniversitat Pompeu Fabra08002BarcelonaSpain; ^9^ Catalan Institution of Research and Advanced Studies (ICREA)08010BarcelonaSpain

**Keywords:** alternative splicing, *Arabidopsis thaliana*, high resolution reverse transcription (HR RT)‐PCR, RNA‐sequencing (RNA‐seq), Sailfish, Salmon, transcripts per million

## Abstract

- RNA‐sequencing (RNA‐seq) allows global gene expression analysis at the individual transcript level. Accurate quantification of transcript variants generated by alternative splicing (AS) remains a challenge. We have developed a comprehensive, nonredundant Arabidopsis reference transcript dataset (AtRTD) containing over 74 000 transcripts for use with algorithms to quantify AS transcript isoforms in RNA‐seq.
- The AtRTD was formed by merging transcripts from TAIR10 and novel transcripts identified in an AS discovery project. We have estimated transcript abundance in RNA‐seq data using the transcriptome‐based alignment‐free programmes Sailfish and Salmon and have validated quantification of splicing ratios from RNA‐seq by high resolution reverse transcription polymerase chain reaction (HR RT‐PCR).
- Good correlations between splicing ratios from RNA‐seq and HR RT‐PCR were obtained demonstrating the accuracy of abundances calculated for individual transcripts in RNA‐seq.
- The AtRTD is a resource that will have immediate utility in analysing Arabidopsis RNA‐seq data to quantify differential transcript abundance and expression.

## Introduction

Alternative splicing (AS) plays a key regulatory role in the growth, development and behaviour of eukaryotic organisms. AS generates multiple transcript isoforms from a gene through variable selection of different splice sites in the precursor mRNA (Black, 2003; Stamm *et al*., 2005; Nilsen & Graveley, 2010). The two main consequences of AS are an increase in proteome complexity by generation of different protein isoforms often with different functionality (Black, 2003; Stamm *et al*., 2005; Nilsen & Graveley, 2010) and the regulation of gene expression through degradation of specific transcripts by the nonsense‐mediated decay (NMD) pathway (Nicholson & Mühlemann, 2010; Kalyna *et al*., 2012; Schweingruber *et al*., 2013).

In higher plants, AS has been implicated in a wide range of developmental and physiological processes (Syed *et al*., 2012; Carvalho *et al*., 2013; Reddy *et al*., 2013; Staiger & Brown, 2013). The functional importance of AS in plants has been illustrated in, for example, organ development, flowering time, and the circadian clock (Zhang & Mount, 2009; Sanchez *et al*., 2010; Airoldi & Davies, 2012; James *et al*., 2012; Jones *et al*., 2012; Posé *et al*., 2013), dark–light retrograde signalling from chloroplast to nucleus (Petrillo *et al*., 2014), and zinc tolerance (Remy *et al*., 2014). AS can be tissue‐specific (Kriechbaumer *et al*., 2012) or regulated by miRNAs and long noncoding RNAs (Jia & Rock, 2013; Bardou *et al*., 2014). The estimated frequency of AS in plants has increased significantly over the last 10 years (Syed *et al*., 2012). RNA sequencing (RNA‐seq) analyses showed that in Arabidopsis at least 61% of intron‐containing genes undergo AS (Filichkin *et al*., 2010; Marquez *et al*., 2012). RNA‐seq permits the genome‐wide identification of all transcript isoforms/splicing variants of a gene and the contribution that each transcript makes to expression. Currently transcript annotation in, for example, The Arabidopsis Information Resource (TAIR), is incomplete, and novel transcript variants continue to be identified in RNA‐seq studies (Filichkin *et al*., 2010; Marquez *et al*., 2012). As the availability of RNA‐seq data grows and AS becomes a routine part of expression analysis, the resolution of gene expression studies will increase and will require accurate methods to quantify transcript isoforms in RNA‐seq. There is a range of available analysis tools that quantify transcripts from reads mapped to a genome or a transcriptome such as Tophat/Cufflinks (Trapnell *et al*., 2009, 2010, 2013; Roberts *et al*., 2011), RSEM (Li & Dewey, 2011; Li *et al*., 2014), eXpress (Roberts & Pachter, 2013), Bayesembler (Maretty *et al*., 2014), and String Tie (Pertea *et al*., 2015). A recent study compared different methods of analysing AS in plant RNA‐seq data and showed variation in their ability to detect and quantify AS events with the accuracy of annotation having a major effect (Liu *et al*., 2014).

Here, we describe the novel application of Sailfish (Patro *et al*., 2014) and Salmon (R. Patro *et al*., unpublished) to Arabidopsis RNA‐seq data. These related programmes are based on lightweight models of sequence alignment and efficient, parallel statistical inference algorithms. Sailfish is a *k*‐mer based method that uses lightweight algorithms to assign *k*‐mers from RNA‐seq reads to transcripts defined in a reference transcriptome (Patro *et al*., 2014). Transcripts are quantified by counting the number of *k*‐mers in RNA‐seq reads and using an expectation‐maximization algorithm to generate individual transcript abundances in transcripts per million (TPM) units (Patro *et al*., 2014). Salmon is based on a novel lightweight alignment model that uses chains of maximal exact matches between sequencing fragments and reference transcripts to determine the potential origin of RNA‐seq reads, and thus eliminates the dependence on a particular, predefined *k*‐mer size required by methods such as Sailfish. Salmon also relies on a novel streaming inference algorithm (an extension of stochastic collapsed variational Bayesian inference; Foulds *et al*., 2013) to improve the accuracy of transcript abundance estimates while maintaining the fast speed and limited memory requirements of Sailfish; it also produces abundance estimates in terms of TPM. In order to use Sailfish and Salmon, we have developed a comprehensive Arabidopsis reference transcript dataset (AtRTD), and have generated quantitative TPM data from RNA‐seq. We have obtained high correlations between data from RNA‐seq and from the high resolution reverse transcription polymerase chain reaction (HR RT‐PCR) system (Simpson *et al*., 2008) with Salmon giving the more accurate quantification. The AtRTD is a resource that will have immediate utility in analysing of Arabidopsis RNA‐seq data to quantify differential transcript abundance using Sailfish, Salmon or other programmes. This approach will be applicable to other plant species dependent on quality and depth of reference transcript dataset construction.

## Materials and Methods

### Construction of AtRTD

To construct the AtRTD, transcripts from The Arabidopsis Information Resource version 10 (TAIR10) (Lamesch *et al*., 2012) and from the AS discovery RNA‐seq analysis, which identified *c*. 50k novel transcripts (Marquez *et al*., 2012), were merged. The AtRTD was refined and developed through iterative validation with HR RT‐PCR data (see the HR RT‐PCR subsection). Redundant transcripts were removed using available tools. First, to remove any confusion caused by differences in the coordinates for the same gene models in the two transcript annotation files, the start and end coordinates of the same gene models in TAIR10 and the AS discovery RNA‐seq analysis (Marquez *et al*., 2012) were compared. Where one was contained within another, the smaller one was removed; where models overlapped partially, the coordinates of the gene model in TAIR10 were retained. Second, the GTF file that describes all the transcript isoforms from the AS discovery RNA‐seq analysis (Marquez *et al*., 2012) was converted to GFF format using perl scripts (http://search.cpan.org/~lds/GBrowse/bin/gtf2gff3.pl version 0.1). The GFF transcript annotation files from the two sources described earlier were then sorted, checked and intron coordinates were extracted using Genome Tools version 1.5.3 (Gremme *et al*., 2013). Thus, when transcripts had the same intron coordinates but differed only in the length of their 5′ or 3′ untranslated regions (UTRs), the longest was retained. Similarly, redundant transcripts from single‐exon genes with different lengths of 5′ or 3′ UTRs were removed. Finally, where transcript fragments occurred, probably due to low read coverage, but had the same intron coordinates of the longer or full‐length transcripts, the fragments were removed. The FASTA file of the merged, nonredundant transcripts was generated using GFFREAD from the Cufflinks tool suite (Trapnell *et al*., 2010). In addition, 33 novel transcripts of core clock genes identified previously (James *et al*., 2012) were also added into the final transcript dataset. These transcripts were identified by HR RT‐PCR; this illustrates that transcripts identified by other methods can be added to the AtRTD although we expect that the need for such manual curation will reduce as the AtRTD develops further. Thus, AtRTD represents a nonredundant, highly comprehensive reference transcript dataset that can be used with transcript quantification programmes. Here, the recently developed Sailfish (*k*‐mer = 30 nt) and Salmon programmes (Patro *et al*., 2014; R. Patro, unpublished) were used in conjunction with the AtRTD to analyse ultra‐deep RNA‐seq data of 5‐wk‐old Arabidopsis plants grown at 20°C and then transferred to 4°C. Three biological samples were harvested at two time‐points: dawn at 20°C (T1) and in the middle of the dark period four days after transfer to the low temperature (T2) for library preparation and sequencing. In total, *c*. 174 and 191 M 100 bp paired end reads were generated for T1 and T2, respectively.

### HR RT‐PCR

To validate the quantification of transcript isoforms, HR RT‐PCR was performed on RNA from the same plant material as RNA‐seq. Primer pairs covering 50 AS events in 29 genes, where the upstream primer was end‐labelled with a fluorescent tag, were used in RT‐PCR reactions with 24 cycles of PCR as described previously (Simpson *et al*., 2008; James *et al*., 2012; Kalyna *et al*., 2012; Marquez *et al*., 2012) and separated on an Illumina 3700 automatic DNA sequencing machine. The abundance of RT‐PCR products was analysed with Genemapper and splicing ratios were calculated from peak areas of each product. We analysed the RNA‐seq data from the same regions and used the TPM values of the transcripts to calculate splicing ratios for comparison with those generated by HR RT‐PCR, following a similar approach to Alamancos *et al*. (2015).

## Results and Discussion

**Figure 1:**
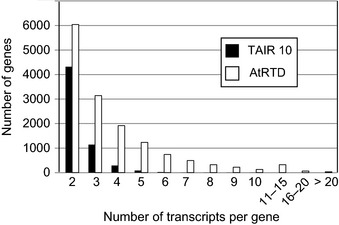
Distribution of the number of transcripts per gene in The Arabidopsis Information Resource version 10 (TAIR10) and the Arabidopsis reference transcript dataset (AtRTD). The number of *Arabidopsis thaliana* genes (*y*‐axis) containing two or more transcripts (*x*‐axis) are shown.

AtRTD was constructed by merging transcripts from TAIR10 and the AS discovery project (Marquez *et al*., 2012). The latter study used a normalized library for RNA‐seq to increase the depth of sequencing and significantly increased the number of transcript isoforms in Arabidopsis despite only using 10‐d‐old seedlings and flower tissue (Marquez *et al*., 2012). The number of genes and transcripts contained in TAIR10, the AS discovery data and AtRTD are shown in Table 1. The gene number in TAIR10 and AtRTD differed by only 23 genes; the lower gene number in Marquez *et al*. (2012) reflects the number of expressed genes in two developmental stages. By contrast, the number of transcripts increased from 41 671 in TAIR10 (that also contains redundant transcripts which differ only by different lengths of 5′ and 3′ UTRs) to 74 216 in the merged and curated AtRTD dataset. The number of transcripts per gene, which reflects transcript isoform complexity, increased from 1.24 in TAIR10, to 2.40 and 2.21 in the AS discovery data and AtRTD, respectively (Table 1). Although the average transcripts per gene values are similar, the number of genes differs significantly (23 905 and 33 625, respectively). The increased transcript complexity in AtRTD compared with TAIR10 is also shown by the number of genes with higher numbers of transcripts (Fig. 1; Supporting Information Table S1). This is illustrated by the reduced number of genes with a single transcript in AtRTD (18 948) compared with TAIR10 (27 717). Thus, AtRTD represents a nonredundant transcript dataset that is highly enriched in AS transcripts. There are 14 499 intron‐containing genes with more than one transcript suggesting that 63.22% of intron‐containing genes undergo AS. It is, however, important to note that because not all developmental stages or environmental conditions are included, it is likely that AS transcripts are still not completely represented and new versions of AtRTD will be generated as other high quality RNA‐seq data becomes available.

**Table 1:** Number of *Arabidopsis thaliana* genes and transcripts in different datasets

	Number of genes	Number of transcripts	Average transcripts per gene
TAIR10	33 602	41 671^a^	1.24
Marquez *et al*. (2012)	23 905	57 408^b^	2.40
AtRTD v3	33 625	74 216^c^	2.21

Note: TAIR10, The Arabidopsis Information Resource version 10; AtRTD, Arabidopsis reference transcript dataset. ^a^Contains redundant transcripts which differ only by different lengths of 5′ and 3′ UTRs. ^b^ *De novo* assembled transcripts defined by splice junctions. ^c^Merged, nonredundant transcripts.

**Figure 2:**
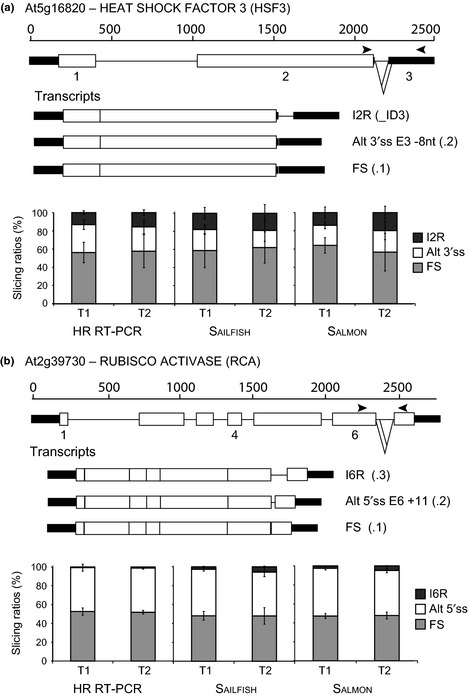
*Arabidopsis thaliana* gene and transcript structures and histograms of transcript ratios from transcripts per million (TPM) generated by Sailfish and Salmon with Arabidopsis reference transcript dataset (AtRTD) and from relative fluorescence units (RFUs) that measures peak areas from high resolution reverse transcription polymerase chain reaction (HR RT‐PCR. (a) At5g16820, *HEAT SHOCK FACTOR 3* (*HSF3*); (b) At2g39730, *RUBISCO ACTIVASE* (*RCA*). Transcripts are shown below the gene structure. Open boxes, exons; black rectangles, untranslated regions; thin lines, introns; diagonal lines, splicing events; arrowheads, approximate positions of primers used in HR RT‐PCR. FS, fully spliced; E, exon; I2R/I6R, intron retention of introns 2 and 6 in (a) and (b), respectively; Alt 3′/5′ss, alternative (a) 3′ and (b) 5′ splice sites, respectively; T1 and T2, different time‐points. Transcript variants from The Arabidopsis Information Resource (TAIR) and the alternative splicing (AS) discovery dataset (Marquez *et al*., 2012) are indicated by .1, .2, or _ID3, respectively. Error bars represent the ± standard deviation (SD) of three independent biological replicates.

**Figure 3:**
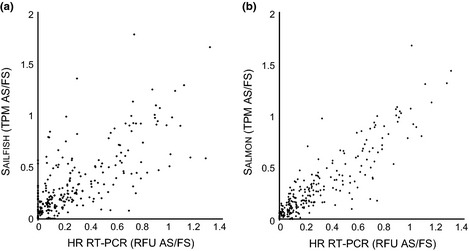
Correlation of the splicing ratios calculated from the RNA‐seq data and the high resolution reverse transcription polymerase chain reaction (HR RT‐PCR. (a) Sailfish, (b) Salmon. Splicing ratios for 50 alternative splicing events from 29 *Arabidopsis thaliana* genes (three biological replicates of the time points T1 and T2) generated 300 data points in total. AS, alternatively spliced; FS, fully spliced; TPM, transcripts per million; RFU, relative fluorescence unit. Pearson's correlation coefficient: Sailfish = 0.7044, Salmon = 0.9051. Spearman's rank correlation coefficient: Sailfish = 0.712, Salmon = 0.907.

To demonstrate the utility of the AtRTD, we have used this set of transcripts to quantify transcripts in RNA‐seq data with the Sailfish and Salmon quantification tools. To validate the quantification of the resulting transcript abundances from RNA‐seq, the TPM of individual transcripts for the genes used in HR RT‐PCR were extracted from the RNA‐seq data. Transcript structures were compared to the AS events covered by the primers in HR RT‐PCR and used to calculate splicing ratios for each of the AS events in that region. The splicing ratio for this comparison is the percentage of transcripts with a particular AS event expressed as a function of the level of total transcripts (Fig. 2). Figure 2 shows histograms of splicing ratios of two gene/AS examples demonstrating the high degree of similarity between the RNA‐seq Sailfish and Salmon outputs and HR RT‐PCR. *HEAT SHOCK FACTOR 3* (*HSF3*) has three different transcripts resulting from AS in a 3′UTR intron: fully spliced, use of alternative 3′ splice site which removes 8 nt from exon 3 and intron 2 retention (Fig. 2a). All three are relatively abundant transcripts and show small differences in splicing ratios at the different time‐points. *RUBISCO ACTIVASE* (*RCA*) undergoes AS in intron 6 (Fig. 2b). An alternative 5′ splice site within the intron adds 11 nt and changes the frame of the resulting protein to give different C‐terminal sequences. Proteins from these two variants differ in length by 28 amino acids and contain eight or 36 different amino acids at their C‐terminal ends. These examples demonstrate that RNA‐seq detects multiple transcripts including transcripts which are present in low abundance (Fig. 2b, I6R), and distinguishes between transcripts differing by a small number of nucleotides (Fig. 2). To directly compare the output from Sailfish and Salmon with HR RT‐PCR, we analysed 50 AS events from 29 genes and three biological replicates of the two time‐points (a total of 300 data points). As in some cases HR RT‐PCR detected relatively low abundance AS transcripts which were not identified in RNA‐seq, for this comparison we calculated a splicing ratio by comparing the abundance of the AS transcript to that of the fully spliced transcript (Fig. 3). For Sailfish and Salmon, the Pearson's correlation coefficient was 0.7044 and 0.9051, respectively, and the Spearman's rank correlation coefficient was 0.712 and 0.907, respectively (Fig. 3).

In this paper we demonstrate that the combination of the AtRTD (a comprehensive nonredundant reference transcript dataset for Arabidopsis) with Sailfish or Salmon allows accurate estimation of individual transcript abundances. The novel AtRTD resource contains a significantly higher number of transcript isoforms than TAIR10 that is still widely used as a reference to analyse RNA‐seq in Arabidopsis. A high degree of correlation between splicing ratios calculated from TPM from RNA‐seq data and the HR RT‐PCR was observed with Salmon outperforming Sailfish. The HR RT‐PCR system was used previously to validate RNA‐seq data qualitatively (Marquez *et al*., 2012) and to monitor changes in AS under different growth conditions and in a range of different mutants (Simpson *et al*., 2008; Raczynska *et al*., 2010; James *et al*., 2012; Jones *et al*., 2012; Kalyna *et al*., 2012; Streitner *et al*., 2012; Petrillo *et al*., 2014) and here we demonstrate its utility in validating quantitative RNA‐seq transcript data. RNA‐seq is now widely used in transcriptome/expression analyses in plants to examine, for example, abiotic and biotic stress responses, development, light regulation and AS itself (e.g. Zhang *et al*., 2010; Rühl *et al*., 2012; Li *et al*., 2013; Ding *et al*., 2014; Thatcher *et al*., 2014; Wu *et al*., 2014; Mandadi & Scholthof, 2015). The AtRTD is freely available for download at http://ics.hutton.ac.uk/atRTD/ and can now be used to analyse such Arabidopsis RNA‐seq data.

## Supporting information

Please note: Wiley Blackwell are not responsible for the content or functionality of any supporting information supplied by the authors. Any queries (other than missing material) should be directed to the *New Phytologist* Central Office.

**Table S1** Transcript complexity of AtRTD: distribution of the number of transcripts per gene
