# Supplementary material for: AtRTD – a comprehensive reference transcript dataset resource for accurate quantification of transcript‐specific expression in Arabidopsis thaliana
Source: New Phytol. 2015 Jun 25;208(1):96–101. doi: 10.1111/nph.13545 (PMC4744958; doi:10.1111/nph.13545)
Supplement: Supplementary file 1 — Table S1 Transcript complexity of AtRTD: distribution of the number of transcripts per gene [file NPH-208-96-s001.pdf]

***New Phytologist* Supporting Information**

Article title: AtRTD - A comprehensive Reference Transcript Dataset resource for accurate quantification of transcript-specific expression in *Arabidopsis thaliana*

Authors: Runxuan Zhang, Cristiane P. G. Calixto, Nikoleta A. Tzioutziou, Allan James, Craig G. Simpson, Wenbin Guo, Yamile Marquez, Maria Kalyna, Rob Patro, Eduardo Eyras, Andrea Barta, Hugh Nimmo and John W. S. Brown

Article acceptance date: 05 June 2015

The following Supporting Information is available for this article:

Table S1 Transcript complexity of AtRTD: Distribution of the number of transcripts per gene

	**No. of genes**	
**No. of transcripts per gene**	**TAIR 10**	**AtRTD**
1	27717	18948
2	4318	6036
3	1144	3142
4	293	1920
5	90	1237
6	26	742
7	7	494
8	5	325
9	1	224
10	1	131
11		112
12		74
13		66
14		35
15		38
16		27
17		9
18		13
19		10
20		6
21		11
22		5
23		4
24		3
25		3
26		2
28		3
29		1
34		1
36		1
40		1
47		1
Total	33602	33625
